# Inter-pregnancy interval and pregnancy outcomes among women with delayed childbearing: protocol for a systematic review

**DOI:** 10.1186/s13643-017-0464-0

**Published:** 2017-04-08

**Authors:** Mani Asgharpour, Sofia Villarreal, Laura Schummers, Jennifer Hutcheon, Dorothy Shaw, Wendy V. Norman

**Affiliations:** 1 Department of Family Practice, University of British Columbia, E204- 4500 Oak Street, Vancouver, BC V6H 3N1 Canada; 2 Department of Epidemiology, Harvard T.H. Chan School of Public Health, 677 Huntington Ave, Boston, MA 02115 USA; 3 Department of Obstetrics and Gynecology, BC Women’s Hospital & Health Centre, University of British Columbia, Shaughnessy C408A, 4500 Oak Street, Vancouver, BC V6H 3N1 Canada; 4 Departments of Obstetrics and Gynecology and Medical Genetics, University of British Columbia, B2 West 4500 Oak Street, Vancouver, BC V6H 3N1 Canada

**Keywords:** Advanced maternal age, Birth interval, Birth spacing, Inter-pregnancy interval, Maternal outcome, Pregnancy complication, Perinatal outcome, Pregnancy spacing

## Abstract

**Background:**

Women in high resource nations are increasingly delaying childbearing until their thirties. Delayed childbearing poses challenges for the spacing of a woman’s pregnancies. Inter-pregnancy intervals <12 months are associated with risk for adverse pregnancy outcome, yet increased maternal age at delivery is linked with increased risk. The optimal inter-pregnancy interval for older mothers is uncertain. This systematic review will aim to assess the relation between inter-pregnancy interval and perinatal and maternal health outcomes in women who delay childbearing to age 30 and older.

**Methods:**

We will search MEDLINE, CINAHL, and EMBASE databases for peer-reviewed articles on the effects of inter-pregnancy interval on perinatal and maternal health outcomes among women over 29 years at the time of first birth, in high-income countries. To assess the quality of studies, the Cochrane’s Collaboration tool for assessing risk of bias will be used for randomized controlled trials, and the Newcastle-Ottawa tool to assess quality of case control and cross-sectional studies. The quality of the findings on each outcome will be assessed across studies, using the GRADE approach. The decision to conduct meta-analyses will be based on the concordance in definitions used for inter-pregnancy intervals, age groups studied, or outcomes measured among selected studies. We will report odds ratios and/or relative risks and/or risk differences for different inter-pregnancy intervals and perinatal and maternal outcomes as well as pregnancy complications.

**Discussion:**

This systematic review will summarize existing data on the relation between inter-pregnancy interval and perinatal and maternal health outcomes among women who delay childbearing to age 30 and older. Findings will inform clinical best practices to assist mothers over age 30 to space their pregnancies appropriately.

**Systematic review registration:**

Prospero CRD42015019057

**Electronic supplementary material:**

The online version of this article (doi:10.1186/s13643-017-0464-0) contains supplementary material, which is available to authorized users.

## Background

There is a growing trend in developed nations for women to delay childbearing to older ages [1–3]. In the USA, the average age of first-time mothers increased 3.6 years from 1970 to 2006, from 21.4 to 25.0 years [1]. The dramatic increase in women having their first birth at the age of 35 years and over has played the largest role in the increased average age of first-time mothers. For example, the US National Center for Health Statistics data indicated that the proportion of first births to women aged 35 years or older increased from one out of 100 in 1970 to one out of 12 in 2006 [1]. Women who delay childbearing are at increased risk of infertility and obstetrical and perinatal complications [4].

As more women have their first birth at older ages compared with several decades ago, they have fewer children and complete their childbearing in a relatively short time span [1]. In one study in the USA, mothers aged 35 years and above at first pregnancy had significantly higher odds of having a short interval between first and second pregnancies compared to mothers of 20–29 years [5]. This is partly due to women being aware of the negative effect of the so-called “biological clock” [6]. As a result, they may be inclined to accelerate subsequent pregnancies in an attempt to minimize the effects of the declining fecundability that is characteristic of advanced maternal age. However, both short (<18 months) and long (>5 years) inter-pregnancy intervals are associated with higher risks of adverse pregnancy outcomes such as preterm birth, low birth weight, and small for gestational age birth [7–9].

Delayed childbearing poses important challenges for planning the spacing of a woman’s pregnancies. At present, the optimal inter-pregnancy interval for women of advanced maternal age at first birth is uncertain. This systematic review study will examine the association between inter-pregnancy interval and perinatal and maternal health outcomes in women age 30 and older at the time of birth, with the aim to provide evidence and recommendations on optimal inter-pregnancy intervals for this particular age group of women.

## Methods

This systematic review protocol adheres to the Preferred Reporting Items for Systematic Review and Meta-Analysis Protocols (PRISMA-P) 2015 statement [10] (Additional file 1) and was registered with the International Prospective Register of Systematic Reviews (PROSPERO) (registration number CRD42015019057).

### Data sources and search strategy

We will conduct computerized searches in MEDLINE, EMBASE, and CINAHL, using a combination of medical subject headings (MeSH) and keywords related to inter-pregnancy interval without any restrictions on time period, language, or study type. A search strategy has been developed in consultation with a research librarian (see Table 1 for the search criteria). The search strategy was piloted across each database to improve the effectiveness of the final search. The bibliographies of all prior systematic reviews and meta-analyses as well as all eligible primary studies will also be reviewed for additional relevant articles. Only peer-reviewed original research articles and conducted in humans will be included. Near the end of the review process, the search will be rerun to identify any potential studies that have been published since the initial search.

**Table 1:** Search strategy

Database	Search term
Medline	Subject heading (MeSH): Birth interval
	Keywords: “birth interval*” or “pregnancy interval*” or “birth spacing*” or “pregnancy spacing*” or “interpregnancy interval*” or “interpregnancy spacing*”
	Birth adj3 Interval
CINAHL	Subject heading (MeSH): Birth interval
	Keywords: “birth interval*” or “pregnancy interval*” or “birth spacing*” or “pregnancy spacing*” or “interpregnancy interval*” or “interpregnancy spacing*”
Embase	Keywords: “birth interval*” or “pregnancy interval*” or “birth spacing*” or “pregnancy spacing*” or “interpregnancy interval*” or “interpregnancy spacing*”
	Birth adj3 Interval

### Eligibility criteria

Studies meeting all of the following criteria will be included: (i) human study, (ii) studies conducted in high resource countries (we will use the definition of “High Income OECD Countries” defined by the World Bank) [11], (iii) studies with analysis or sub-analyses of results among women age 30 or older, and (iv) studies on the relationship between inter-pregnancy interval and perinatal, maternal, or pregnancy health outcomes. The primary outcomes are perinatal health outcomes (preterm birth, low birth weight, small for gestational age, stillbirth, NICU admission, neonatal and infant mortality). Secondary outcomes are (i) maternal health outcomes (cesarean delivery, uterine rupture, maternal ICU admission, severe maternal morbidity, or maternal mortality), (ii) pregnancy complications (preeclampsia, gestational diabetes), and (iii) complications of labor and delivery (dystocia, post-partum hemorrhage).

### Study selection and data management

All papers identified from the initial electronic search process will be imported into a Refworks library [12], and duplicates will be removed. Titles and abstracts will be screened by two investigators (MA and SV). Discrepancies will be resolved through consultation with a third reviewer (WN). Following the screening process, the full text of potentially eligible studies will be retrieved. Two independent reviewers will screen at the full text stage according to the eligibility criteria. Any discrepancies between the two reviewers for included or excluded studies will be discussed, and if an agreement cannot be reached, two senior reviewers will be used to reach consensus (JH and WN). The reason for excluding each study will be recorded. At this stage, the reference lists of included studies will be scanned, and if any relevant studies are identified, the full text will be retrieved and reviewed for inclusion by both reviewers. We will decide whether to conduct a meta-analysis based on whether the individual studies differed considerably in definitions used for inter-pregnancy intervals, age groups studied, or outcomes measured. We will report odds ratios and/or relative risks and/or risk differences for different inter-pregnancy intervals and perinatal and maternal outcomes as well as pregnancy complications. We will document whether eligible studies have controlled for, or otherwise taken into consideration, potential confounders, such as socioeconomic status, pre-existing medical conditions, previous gynecological, or obstetrical history, while examining the relationship between inter-pregnancy interval and perinatal, maternal, or pregnancy health outcomes.

### Quality assessment

The quality of studies included in this review will be assessed by two researchers (WN and MA) using a tool appropriate for the study design. Any discrepancies between the two reviewers will be discussed, and if a consensus on study quality rating cannot be reached, advice will be sought from a third reviewer (JH). For RCTs, Cochrane’s Collaboration tool for assessing risk of bias in randomized trials [13] will be used. This tool includes six domains to assess bias (i.e., selection bias, performance bias, detection bias, attrition bias, and reporting bias) which are assigned as either “low risk of bias,” “unclear risk of bias,” or “high risk of bias” [13]. This information will be presented as a risk of bias summary figure. To assess the study quality of prospective and cross-sectional studies, the Newcastle-Ottawa Scale (NOS) for cohort studies will be used [14]. This tool assigns stars to indicate higher quality based on three broad criteria, specific to the study design (i.e., selection of study groups, comparability and outcome assessment). This information will be presented in a summary table, indicating the star rating for each individual study included in the review. For all studies included in this review, the information on effect size will be recorded and assessed. Effect size will be either extracted from the study or calculated if the study does not report the information, using the mean values and standard deviation retrieved from the study.

Besides the quality assessment of individual studies, we will also assess the quality of the findings on primary and secondary outcomes across studies, using GRADE guidelines, which were developed by the Grading of Recommendations, Assessment, Development and Evaluations (GRADE) Working Group and adopted by BMJ Clinical Evidence [15]. The GRADE approach will allow us to consider multiple key factors to determine the quality of the evidence of each outcome, and therefore help appraise how confident we are in the body of evidence [16]. A Summary of Findings table will be generated to present the quality of the evidence, the magnitude of the effect, and reasons behind decisions.

## Discussion

This will be the first systematic review to examine the association between inter-pregnancy interval and perinatal and maternal health outcomes in women age 30 and older at the time of birth. While it is already recognized that both short and long inter-pregnancy intervals increase the risk of adverse pregnancy outcomes [7, 8], a greater understanding of these effects in women who delay childbearing to age 30 and older would provide clinical practitioners and mothers a better knowledge base for decision-making.

## Additional file

Additional file 1: PRISMA-P 2015 checklist. (DOCX 70 kb)

## Abbreviations

CINAHL: Cumulative Index to Nursing and Allied Health Literature
EMBASE: Excerpta Medica database
MEDLINE: Medical Literature Analysis and Retrieval System Online
